# Editorial: POSEIDON’s Stratification of ‘Low Prognosis’ Patients in ART: The WHY, the WHAT, and the HOW

**DOI:** 10.3389/fendo.2021.719647

**Published:** 2021-06-29

**Authors:** Sandro C. Esteves, Claus Yding Andersen, Robert Fischer, Peter Humaidan, Carlo Alviggi

**Affiliations:** ^1^ ANDROFERT, Andrology and Human Reproduction Clinic, Campinas, Brazil; ^2^ Faculty of Health, Aarhus University, Aarhus, Denmark; ^3^ Laboratory of Reproductive Biology, Faculty of Health and Medical Sciences, University Hospital of Copenhagen, Copenhagen, Denmark; ^4^ Fertility Center Hamburg, Hamburg, Germany; ^5^ Fertility Clinic Skive, Skive Regional Hospital, Skive, Denmark; ^6^ Department of Neuroscience, Reproductive Science and Odontostomatology, University of Naples, Federico II, Naples, Italy

**Keywords:** assisted reproductive technology, infertility, POSEIDON criteria, ovarian stimulation, low prognosis, poor ovarian response, editorial

Management of patients with infertility and poor or suboptimal ovarian response to exogenous gonadotropin stimulation has challenged reproductive specialists for a long time. Apart from the limited understanding of its pathophysiology, there is wide heterogeneity in the definition of poor responders and overall disappointing outcomes when these patients undergo assisted reproductive technology (ART).

The **P**atient-**O**riented **S**trategies **E**ncompassing **I**ndividualize**D O**ocyte **N**umber (POSEIDON) criteria were introduced in 2016 with the primary goal of underlining differences related to a poor or suboptimal infertility treatment outcome in terms of oocyte quantity and quality, and possibly creating more homogenous groups for clinical management and research (1, 2). The POSEIDON criteria classify patients with infertility undergoing ART into four groups of ‘low-prognosis’ based on female age, ovarian reserve markers (antral follicle count and/or anti-Mullerian hormone), and the number of oocytes retrieved after a standard ovarian stimulation (**Figure 1**). By contrast, patients with adequate ovarian reserve markers and normal response to ovarian stimulation (>9 oocytes retrieved) can be classified as having a ‘normal’ prognosis (non-POSEIDON patients).

**Figure 1:**
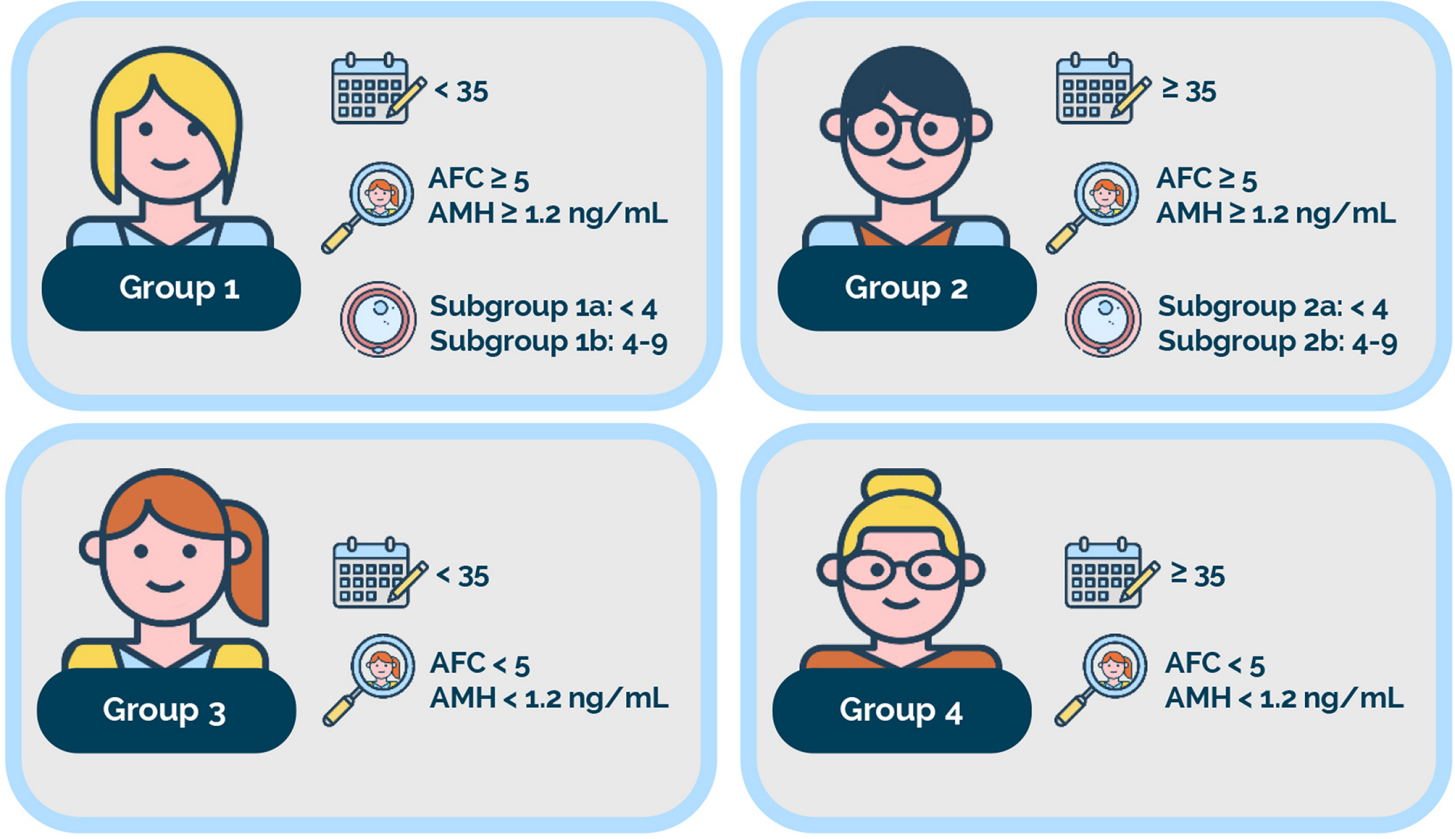
POSEIDON criteria. Four distinct groups of low-prognosis patients can be established based on quantitative and qualitative parameters, namely: 1. The age of the patient and its related embryo aneuploidy rate; 2. Ovarian biomarkers [antral follicle count (AFC) and/or anti-Müllerian hormone (AMH)], and 3. The ovarian response in terms of oocyte quantity (if a previous cycle of conventional ovarian stimulation was carried out). Group 1: Patients <35 years with sufficient pre-stimulation ovarian reserve parameters (AFC ≥5, AMH ≥1.2 ng/ml) and with an unexpected poor (<4 oocytes) or suboptimal (four to nine oocytes) ovarian response. This group is further divided into subgroup 1a, constituted by patients with fewer than four oocytes; and subgroup 1b, constituted by patients with four to nine oocytes retrieved after standard ovarian stimulation, who, at any age, have a lower cumulative delivery rate than age-matched normal responders. Group 2: Patients ≥35 years with sufficient pre-stimulation ovarian reserve parameters (AFC ≥5, AMH ≥1.2 ng/ml) and with an unexpected poor or suboptimal ovarian response. This group is further divided into subgroup 2a, constituted by patients with fewer than four oocytes; and subgroup 2b, constituted by patients with four to nine oocytes retrieved after standard ovarian stimulation, who, at any age, have a lower cumulative delivery rate than age matched normal responders. Group 3: Patients <35 years with poor ovarian reserve pre-stimulation parameters (AFC <5, AMH <1.2 ng/ml). Group 4: Patients ≥35 years with poor ovarian reserve pre-stimulation parameters (AFC <5, AMH <1.2 ng/ml). Art drawing courtesy of Chloé Xilinas, Med.E.A., Rome, Italy. Reprint from: Copyright ^©^ 2021 Esteves SC, Conforti A, Sunkara SK, Carbone L, Picarelli S, Vaiarelli A, Cimadomo D, Rienzi L, Ubaldi FM, Zullo F, Andersen CY, Orvieto R, Humaidan P and Alviggi C (2021) Improving Reporting of Clinical Studies Using the POSEIDON Criteria: POSORT Guidelines. Front. Endocrinol. 12:587051. This is an open-access article distributed under the terms of the Creative Commons Attribution License (CC BY). The use, distribution or reproduction in other forums is permitted, provided the original author(s) and the copyright owner(s) are credited and that the original publication in this journal is cited, in accordance with accepted academic practice. No use, distribution or reproduction is permitted which does not comply with these terms.

POSEIDON patients are presumed to be at a higher risk of failing to achieve a live birth after *in vitro* fertilization/intracytoplasmic sperm injection (IVF/ICSI) treatment than non-POSEIDON patients for two main reasons, namely, reduced number of oocytes and, consequently, embryos; and poor oocyte/embryo quality, due to advanced female reproductive age (3–6).

Given its novelty and potential clinical and research utility, we developed a Research Topic fully dedicated to the POSEIDON criteria. We aimed to provide clinicians and scientists involved in the study and care of infertile couples a thoughtful and comprehensive review of the significance of the POSEIDON concept and its implications for practice and research. This Frontiers Research Topic on ‘POSEIDON Stratification of Low-Prognosis Patients in ART: The WHY, the WHAT, and the HOW’ comprises the seminal work of 65 renowned clinicians, embryologists, and scientists from 30 Institutions and fifteen countries on four continents. In 21 articles, authoritative reviews, original articles, and commentaries dissect the POSEIDON criteria from various angles, including epidemiology, pathophysiology, genetics, ovarian biomarkers, ovarian stimulation strategies, and other treatment modalities.

The first section (The ‘WHY’) explains the reasons why the POSEIDON criteria were developed. Five articles (Esteves, Roque et al., Grisendi et al., Cimadomo et al., Alviggi, Conforti et al., Grynberg and Labrosse) clarify this aspect and provide further insights into parameters used to classify patients fulfilling the POSEIDON criteria. One of them goes further by proposing a new maker –termed Follicle-to-Oocyte (FOI) Index– to identify the patients with ovarian hyporesponse to exogeneous gonadotropin stimulation (Alviggi, Conforti et al.).

The second section (The ‘WHAT’) comprises articles (Esteves, Alviggi et al., Esteves, Carvalho et al., Esteves, Yarali, Ubaldi et al., Fischer and Baukloh, Esteves and Carvalho) explaining in detail what the POSEIDON criteria are and their potential clinical implications for the diagnosis and management of infertility. Two of them (Esteves, Carvalho et al., Esteves, Yarali, Ubaldi et al.) are original articles, related to the development and validation of a novel predictive model to estimate the number of metaphase II (MII) oocytes required to obtain at least one euploid blastocyst for transfer in couples undergoing IVF/ICSI. Notably, the ability to retrieve the number of oocytes needed to achieve at least one euploid embryo for transfer was proposed by the POSEIDON group as an intermediate marker of a successful outcome in IVF/ICSI cycles. The predictive model mentioned above was the backbone of the so-called ‘ART calculator’. It is an online tool that makes two types of predictions, one using pretreatment information to estimate the minimum number of MII oocytes to achieve ≥1 euploid blastocyst, and another based on the actual number of mature oocytes collected/accumulated to estimate the chances of having a euploid blastocyst using that oocyte cohort for IVF/ICSI. The novel ART calculator may assist in clinical counseling and individualized treatment planning regarding the number of oocytes required for at least one euploid blastocyst in IVF/ICSI procedures, all aspects debated in dedicated commentaries (Fischer and Baukloh, Esteves and Carvalho).

Another original article within section two relates to a multicenter and multinational prevalence study of more than 13,000 patients who have undergone ART (Esteves, Yarali, Vuong et al.). This article confirms that POSEIDON patients are very common in the Fertility Clinic, representing about 43.0% of all treated patients. In this study, most POSEIDON patients were poor (<4 oocytes retrieved) or suboptimal (4-9 oocytes retrieved) responders despite having adequate ovarian reserve markers (Groups 1 and 2), thus highlighting opportunities for refining the clinical management of this vulnerable patient population. Additionally, this big data study showed that POSEIDON patients were older, had a higher body mass index, lower ovarian reserve markers, and a higher frequency of female factor as the primary treatment indication than non-POSEIDON patients, i.e, those patients not fulfilling the POSEIDON criteria. Lastly, this study showed that POSEIDON patients required larger doses of gonadotropin for ovarian stimulation, despite achieving a 2.5 times lower number of retrieved oocytes than normal responders with adequate ovarian markers (non-POSEIDON patients).

The last part (The ‘HOW’) is devoted to the clinical management of POSEIDON patients and how to conduct research using the POSEIDON classification. This section contains ten articles, including reviews (Conforti et al., Haahr et al.), original papers (Drakopoulos et al., Vaiarelli et al.), commentaries (Polyzos and Drakopoulos, Bühler, Sunkara et al., Fischer and Baukloh), future perspectives (Humaidan et al.), and guidelines (Esteves, Conforti, Sunkara et al.). The latter ‘Policy and Practice Reviews’ article (Esteves, Conforti, Sunkara et al.) is timely because the number of published studies using the POSEIDON criteria has increased steadily; however, inconsistent and incomplete reporting of critical outcomes are commonly seen.

At the time of writing (May 21), the entry ‘POSEIDON criteria’ on PubMed retrieved more than 50 articles of all sorts. Failure to recognize the critical pillars of the POSEIDON criteria, as mentioned above, might limit the clinical utility of such studies, notably when the essential endpoints are incompletely reported –or not reported at all. With this in mind, the ‘Improving Reporting of Clinical Studies Using the POSEIDON Criteria’ (POSORT) guidelines aim at help researchers improve the quality of reporting in studies applying the POSEIDON classification system (Esteves, Conforti, Sunkara et al.).

Other studies outside this Research Topic have substantiated the validity of the POSEIDON criteria in identifying relevant subpopulations with low-prognosis in IVF/ICSI treatment. It has recently been reported that either AFC or AMH can be used as the ovarian marker criterion for patient classification within the context of POSEIDON (7). Based on 9484 patients whose baseline ovarian reserves had been assessed by both AFC and AMH, a strong agreement (kappa~0.8) between AMH and AFC was found to classify POSEIDON patients. Approximately 75% of individuals were classified under the same patient group using both biomarkers. Importantly, virtually all patients with discordant biomarker results remained within the broad category of ‘low prognosis’ as defined by the POSEIDON criteria. Another finding of the study was that the optimal AFC and AMH (by Gen II assay) thresholds to predict the retrieval of <4 oocytes were 5 and 1.27 ng/mL, respectively, and thus like those established by the POSEIDON criteria (see **Figure 1**). Also, for the first time AFC and AMH thresholds were provided to identify suboptimal responders, i.e., patients who end up with an oocyte yield between 4 and 9 after standard ovarian stimulation. An AFC of 12 and an AMH value of 2.95 ng/mL (Gen II assay) were the optimal thresholds below which the risk of retrieving a ‘suboptimal’ (4-9 oocytes) oocyte number after standard ovarian stimulation is increased. Collectively, this study (7) showed that both biomarkers provide acceptable and equivalent accuracy in predicting oocyte yield further supporting their use and proposed thresholds in daily clinical practice for patient classification according to the POSEIDON criteria.

As previously stated, POSEIDON patients are presumed to be at a higher risk of failing to achieve a live birth after IVF/ICSI than normal responders with an adequate ovarian reserve. The cumulative delivery rate (CDR) per initiated/aspiration cycle after the transfer of all fresh and frozen–thawed/warmed embryos has been suggested to be the critical endpoint that sets these groups apart. This metric is increasingly recognized as an appropriate way to report ART success (8, 9) and has been selected as a critical efficacy outcome marker in the ESHRE 2019 guideline on ovarian stimulation for IVF/ICSI (10). It is considered the most meaningful outcome from the patients’ perspective because it adequately reflects the prognosis of achieving a live birth after one initiated/aspirated ART cycle (11). Along these lines, a 2021 multinational study including over 9,000 patients showed that the CDR is, on average, ~50% lower in POSEIDON patients than non-POSEIDON patients, and it varied across POSEIDON groups (12). Interestingly, the CDR was twice as high in suboptimal responders (4-9 oocytes retrieved) as in poor responders (<4 oocytes), an effect that was primarily modulated by female age. Furthermore, logistic regression analysis showed that the POSEIDON stratification, the number of embryos obtained, the number of embryo transfer cycles per patient, the number of oocytes retrieved, female age, duration of infertility, and body mass index were relevant predictors of CDR (12).

This Research Topic has a broad appeal, and we hope it stimulates further research in terms of early diagnosis, prevention, and identification of specific interventions that could benefit POSEIDON patients. We, as guest editors, are grateful to the Chief Editors and the Editorial staff of Frontiers in Endocrinology (Reproduction) for their outstanding support. We recommend this Research Topic to clinicians involved in the management of infertile couples, including reproductive endocrinologists, gynecologists, reproductive urologists, andrologists, embryologists, as well as other healthcare professionals providing care to infertility patients. Also, students and researchers in the biological and medical sciences, interested in following the exponential growth in knowledge involving female infertility and ART might greatly benefit from this collection of articles. We hope our readers will appreciate this Frontiers Research Topic and that they share our excitement in studying infertility and assisted reproductive technology.

## Author Contributions 

All authors contributed to the article and approved the submitted version.

## Funding

The publishing fees of the articles included in this Research Topic were funded by an unrestricted educational grant for Merck, Darmstadt.

## Conflict of Interest

SE and CA declare receipt of unrestricted research grants from Merck and lecture fees from Merck and Med.E.A. PH has received unrestricted research grants from MSD, Merck, and Ferring as well as honoraria for lectures from MSD, Merck, Gedeon–Richter, Theramex, IBSA, and Med.E.A. CA has received unrestricted grants from Gedeon-Richter and honoraria for lectures from IBSA, Ferring, and Merck. RF has received lecture fees from Merck and Med.E.A. All authors are co-founders of the POSEIDON group.

## References

[1] Alviggi C Andersen CY Buehler K Conforti A De Placido G Esteves SC. A New More Detailed Stratification of Low Responders to Ovarian Stimulation: From a Poor Ovarian Response to a Low Prognosis Concept. Fertil Steril (2016) 105:1452–3. 10.1016/j.fertnstert.2016.02.005 26921622

[2] Humaidan P Alviggi C Fischer R Esteves SC. The Novel Poseidon Stratification of ‘Low Prognosis Patients in Assisted Reproductive Technology’ and Its Proposed Marker of Successful Outcome. F1000Res (2016) 5:2911. 10.12688/f1000research.10382.1 28232864 PMC5302217

[3] Sunkara SK Rittenberg V Raine-Fenning N Bhattacharya S Zamora J Coomarasamy A. Association Between the Number of Eggs and Live Birth in IVF Treatment: An Analysis of 400 135 Treatment Cycles. Hum Reprod (2011) 26:1768–74. 10.1093/humrep/der106 21558332

[4] Esteves SC Carvalho JF Martinhago CD Melo AA Bento FC Humaidan P. Estimation of Age-Dependent Decrease in Blastocyst Euploidy by Next Generation Sequencing: Development of a Novel Prediction Model. Panminerva Med (2019) 61:3–10. 10.23736/S0031-0808.18.03507-3 29962186

[5] Drakopoulos P Blockeel C Stoop D Camus M de Vos M Tournaye H. Conventional Ovarian Stimulation and Single Embryo Transfer for IVF/ICSI. How Many Oocytes Do We Need to Maximize Cumulative Live Birth Rates After Utilization of All Fresh and Frozen Embryos? Hum Reprod (2016) 31:370–6. 10.1093/humrep/dev316 26724797

[6] Esteves SC Roque M Sunkara SK Conforti A Ubaldi FM Humaidan P. Oocyte Quantity, As Well as Oocyte Quality, Plays a Significant Role for the Cumulative Live Birth Rate of a POSEIDON Criteria Patient. Hum Reprod (2019) 34(12):2555–7. 10.1093/humrep/dez181 31756248

[7] Esteves SC Yarali H Vuong LN Carvalho JF Özbek İY Polat M. Antral Follicle Count and Anti-Müllerian Hormone to Classify Low-Prognosis Women Under the POSEIDON Criteria: A Classification Agreement Study of Over 9000 Patients. Hum Reprod (2021) 36(6):1530–41. 10.1093/humrep/deab056 33822057

[8] Maheshwari A McLernon D Bhattacharya S. Cumulative Live Birth Rate: Time for a Consensus? Hum Reprod (2015) 30:2703–7. 10.1093/humrep/dev263 26466912

[9] Zegers-Hochschild F Adamson GD Dyer S Racowsky C de Mouzon J Sokol R. The International Glossary on Infertility and Fertility Care, 2017. Hum Reprod (2017) 32:1786–801. 10.1093/humrep/dex234 PMC5850297 29117321

[10] Ovarian Stimulation TEGGO Bosch E Broer S Griesinger G Grynberg M Humaidan P. Eshre Guideline: Ovarian Stimulation for IVF/ICSI†. Hum Reprod Open (2020) 2020:hoaa009. Erratum in: Hum Reprod Open (2020) 2020:hoaa067. 10.1093/hropen/hoaa009 32395637 PMC7203749

[11] Malizia BA Hacker MR Penzias AS. Cumulative Live-Birth Rates After *In Vitro* Fertilization. N Engl J Med (2009) 360:236–43. 10.1056/NEJMoa0803072 19144939

[12] Esteves SC Yarali H Vuong LN Carvalho JF Özbek İY Polat M. Cumulative Delivery Rate Per Aspiration *In Vitro* Fertilization/Intracytoplasmic Sperm Injection Cycle in POSEIDON Patients: A Real-World Evidence Study of 9073 Patients. Hum Reprod. 10.1093/humrep/deab152 PMC8289325 34179973

